# Sex differences of language abilities of preschool children with autism spectrum disorder and their anatomical correlation with Broca and Wernicke areas

**DOI:** 10.3389/fped.2022.762621

**Published:** 2022-07-22

**Authors:** Yun Zhang, Bin Qin, Longlun Wang, Jie Chen, Jinhua Cai, Tingyu Li

**Affiliations:** ^1^National Clinical Research Center for Child Health and Disorders, Ministry of Education Key Laboratory of Child Development and Disorders, Chongqing Key Laboratory of Translational Medical Research in Cognitive Development and Learning and Memory Disorders, Department of Radiology, Children's Hospital of Chongqing Medical University, Chongqing, China; ^2^Chongqing Engineering Research Center for Clinical Big Data and Drug Evaluation, Medical Data Science, Academy of Chongqing Medical University, Chongqing, China; ^3^Children Nutrition Research Center, Children's Hospital of Chongqing Medical University, Chongqing, China

**Keywords:** autism spectrum disorder, language, preschool children, Broca area, Wernicke area

## Abstract

**Objective:**

People with autism spectrum disorder (ASD) often have language difficulties. This study focuses on whether there are sex differences in language ability in children with ASD and aims to analyze whether such differences may arise from developmental imbalances in the anatomical structures of Broca and Wernicke areas.

**Methods:**

The language development quotient (DQ) scores of Gesell Developmental Scale (GDS) and the scores of language communication of Childhood Autism Rating Scale (CARS) were used to judge the language ability, and the FREESURFER software extracted the anatomical structures of Broca and Wernicke areas on 3DT1 sequences. We analyzed the correlation between the anatomical structure of Broca/Wernicke areas and language abilities assessments.

**Results:**

The study initially included 44 cases of ASD, with 36 males (81.8 %) and 8 females (18.2%), and the age range was 24–72 months. Males have better language abilities than females. Specifically, the GDS verbal DQ of males was significantly higher than that of females (56.50 ± 18.02 vs. 29.23 ± 6.67, *p* < 0.001). Broca thickness*-L* was positively correlated with verbal DQ scores in GDS (*r* = 0.382, *p* = 0.011) and lower than grade 2 and 3 on the CARS verbal communication grade 4 (5.76 ± 0.17 vs. 6.21 ± 0.30 and 6.11 ± 0.35), with statistically significant differences between groups (*p* < 0.05).

**Conclusion:**

There were sex differences in the language abilities of preschoolers with ASD, which may be due to an imbalance development of certain structures in Broca and Wernicke areas, especially Broca area.

## Introduction

People with autism spectrum disorder (ASD) often have language difficulties. Approximately 25–30% of children with ASD either fail to develop functional language or are minimally verbal (1), because of which they often draw the attention of their guardians and society. The language ability is also considered to be an indicator of the prognosis of ASD (2). It has been found that children with ASD who have high initial language abilities improve their abilities over time, while children with low initial language abilities do not show significant improvements in most language indicators at follow-up (3). The literature reports that most children with ASD experience language delays, and although by school age many children have caught up with their peers, some children have been found to continue to lag behind their peers, with significant impairments in both expressive and receptive language (4, 5).

The structure of language center is complex, and there are sex differences in clinical symptoms in children with ASD. Clinical data suggest that girls with ASD present more abnormalities in sensory profile. Girls with ASD are at greater risk for developing anxiety, depression, suicidal ideation, and psychiatric hospitalization. Boys with ASD appear to be at greater risk for cooccurring attention-deficit and hyperactivity disorder, obsessive-compulsive disorder, and tic syndrome (6). Also, significant sex differences were found in narrative production. Girls with ASD included more salient story elements than boys (7). There are relatively few studies on the structure of language center in ASD, and the choice of language center and findings is inconsistent, with more researchers choosing the temporal plane to study. The analysis by Lombardo et al. (8) using task-state fMRI showed that young people with ASDs aged 3–4 with good language levels had higher activation in the temporal plane, whereas young people with ASDs with poor language scores had lower activation. Studies have also found significant structural asymmetries in the temporal plane of the bilateral cerebral hemispheres in people with language impairment (9). Knaus et al. (10) suggested that the volume of the inferior frontal gyrus triangle and temporal plane is significantly reduced in people with severe language impairment. Lee et al. (11) found that children and adolescents with ASD had reduced degree centrality (DC) in the Broca area, whereas adults with ASD had reduced DC in the Wernicke areas. Verly et al. (12) found that the connectivity between Broca area and Wernicke area remained in patients with ASD, but that the connectivity between Broca area and the prefrontal area was significantly reduced between the two hemispheres, and that the functional connectivity between the right cerebellum and the language center was significantly reduced.

Since our previous study (13) showed that the anterior-middle corpus callosum (AMCC) and mid-posterior corpus callosum (PMCC) volumes were higher in children with ASD compared to controls, and since interhemispheric connections in the Broca area of the forebrain rely mainly on the AMCC according to the Brodmann brain subdivision system, while interhemispheric connections in the Wernicke area of the hindbrain rely mainly on the PMCC, we hypothesized that the changes in corpus callosum (CC) volumes in children may be related to the overgrowth of efferent fibers in the language center. We also found that the majority of children with ASD showed mainly impaired language abilities, whereas a small number had moderate or even high expressive language abilities. Because preschool is a critical period for language formation, we analyzed the language abilities of preschool children with ASD in relation to the anatomy of Broca and Wernicke areas and provided some guidance for later early intervention.

## Materials and methods

### Ethical approval

This study was approved by the Research Ethics Committee of Children's Hospital of Chongqing Medical University (IRB number: [2018] ethical review [research] no. [82]). All parents gave written informed consent after they had been informed of the possible risks and benefits of the research and assured of the security and privacy concerning the children's medical records.

### Participants

All participants were recruited at the child healthcare department of Children's Hospital of Chongqing Medical University, a tertiary hospital, and National Children's Medical Center in China, from December 2020 to January 2021. The clinical assessment of children with ASD includes an initial assessment of ASD using the Social Responsiveness Scale and Autism Behavior Checklist instruments, further clinical confirmation of ASD using the Diagnostic and Statistical Manual of Mental Disorders, Fifth Edition (14) and a sample test using the Autism Diagnostic Observation Schedule, Second Edition as a quality control. Patients with ASD were included if their age was between 2 and 6 years, were right-handed, and had no significant abnormalities on routine MRI sequences. Exclusion criteria included other neurological disorders, genetic conditions, structural brain abnormalities, and the presence of any MRI contraindications (e.g., metal implants, braces, claustrophobia). This is a prospective study.

### Clinical features assessment

Self-administered general condition scales were used to obtain general demographic information and medical history of all subjects. The Gesell Development Scale (GDS) was completed by a homogeneously trained physician on the day of the examination and when the child was not sedated. The GDS consists of five functional areas, namely, adaptive, gross motor, fine motor, language, and personal social skills, and the language development quotient (DQ) scores were extracted for analysis. Based on the language DQ scores, there were five levels, with DQ > 75 classified as normal group, 55 ≤ DQ ≤ 75 as mild intellectual disability, 40 ≤ DQ ≤ 54 as moderate intellectual disability, 25 ≤ DQ ≤ 39 as severe intellectual disability, and DQ < 25 as very severe intellectual disability (10).

The Childhood Autism Rating Scale (CARS) scale is one of the most widely used tests and rating scales for autism, applicable to children over 2 years of age, with good reliability, validity, and practicality. The CARS scale consists of 15 rating items, each of which is divided into four scales, namely, 1, 2, 3, and 4. This study focused on analyzing the scores of language communication. The significance of the levels of language communication is as follows: 1, age-appropriate; 2, mildly abnormal; 3, moderately abnormal; and 4, severely abnormal (15).

### MRI data acquisition

After assessment of the situation by the anesthesiologist, intravenous propofol sedation was pushed at an induction dose of 3 mg/kg, followed by continuous propofol Target-Controlled Infusion (TCI) pumping. Each subject's MR data were obtained by a 3 Tesla MR-scanner (GE Discovery MR 750) with an eight-channel head coil. The child's head was fixed with a sponge cushion before scanning. Each case underwent routine MR scan with the sequences including axial T1WI, T2W1, T2FLAIR, and sagittal T2WI, to exclude obvious organic neurological disease. After routine MR scan, a sagittal 3DT1 BRAVO sequence was performed. The parameters of the sequence were as follows: repetition time (TE) = 3.19 ms, echo time (TE) = 8.22 ms, inversion time TI (T1) = 450 ms, bandwidth (BW) = 122 Hz, layer thickness = 1 mm, interva*l* = 0, flip angle = 12°, FOV. Total scan time was 4 min 56 s, with a total of 164 layers.

### MRI analysis

Routine sequence images were analyzed and reported by two attending physicians and confirmed by a professor from the radiology department. Postprocessing of the original 3D-BRAVO images was performed by a professionally trained attending physician. The analysis was performed using FREESURFER v7.0 (http://surfer.nmr.mgh.harvard.edu/) to extract the cortical thickness, surface, and volume of the corresponding brain areas on 3DT1 sequences. The FREESURFER software is well-documented and freely available for download. The technical details of these procedures are described in Fischl (2012) (16). FREESURFER is a structural model of the human brain created on the basis of 3D-SPGR images using a series of algorithms to quantify the structure as well as the function of the brain, allowing for 3D reconstruction and spreading the images to automatically extract and calculate structural indicators of the cerebral cortex (cortical thickness, surface area, and volume). Briefly, this processing consisted of two main stages, namely, volumetric processing and surface processing.

**Figure 1:**
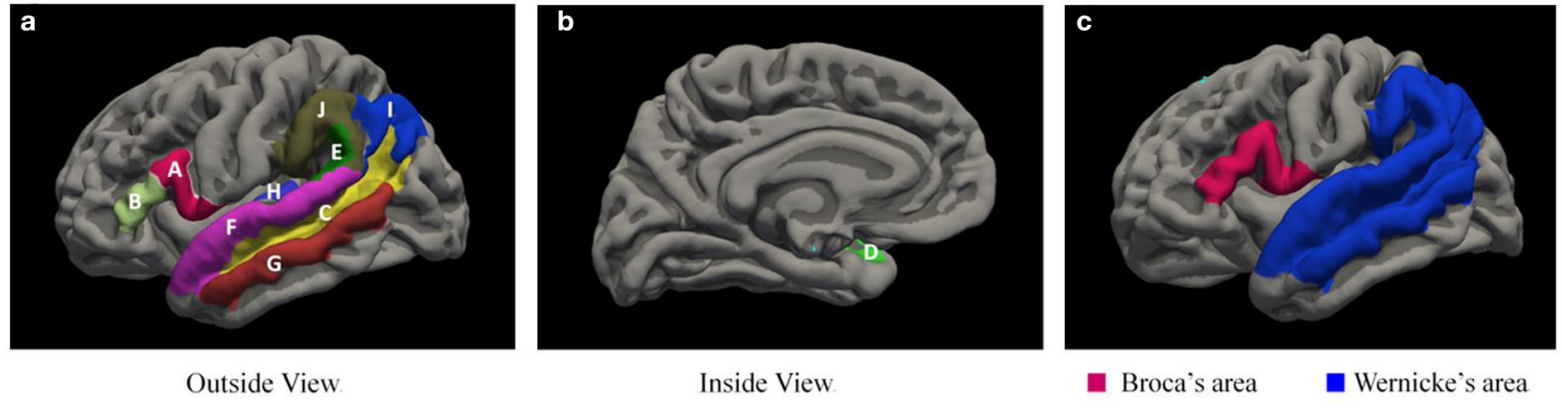
Language*-r*elated brain areas extracted by the FREESURFER V7.0 software. **(a,b)** shows two brain areas contained by Broca area and eight brain areas contained in Wernicke area, and **(c)** shows the overall extent of Broca area and Wernicke area. A, inferior frontal gyrus pars opercularis; B, inferior frontal gyrus pars triangularis; C, Heschl's gyrus; D, planum polare; E, planum temporale; F, superior temporal gyrus; G, middle temporal gyrus; H, superior temporal sulcus; I, angular gyrus; J, supramarginal gyrus.

The delineation of Broca area is currently relatively clear, containing the Brodmann areas 44 and 45, whereas there is no consensus on the delineation of some of the boundaries of Wernicke area (17–20), and it is still controversial whether structures such as the superior temporal sulcus, for example, are delineated as Wernicke area. In this study, we have included the superior temporal sulcus in the Wernicke area by referring to related studies (20–22). Due to the number of anatomical structures contained in Broca and Wernicke areas, we combined the corresponding brain areas for a clearer statistical analysis and finally analyzed only the cortical thickness, surface, and volume in Broca and Wernicke areas, with cortical thickness taken as the average of the thickness of each area and surface area and volume taken as the sum of the areas. A total of 10 brain areas contained in Broca and Wernicke areas (17–20) were extracted in bilateral cerebral hemispheres using the FREESURFER v7.0 (16) software (Figure 1).

The cortical thickness of the Broca area and Wernicke area is the average of the thickness of each area, while the surface and volume is the sum of the areas. The specific calculation method is as follows:

Broca thickness-L =(A + B)/2;
Broca thickness-R =(A + B)/2;
Broca surface-L = C + D + E + F + G + H + I + J;
Broca surface-R = C + D + E + F + G + H + I + J;
Broca volume-L = C + D + E + F + G + H + I + J;
Broca volume-R = C + D + E + F + G + H + I + J;
Wernicke thickness-L =(A + B)/2;
Wernicke thickness-R =(A + B)/2;
Wernicke surface-L = C + D + E + F + G + H + I + J;
Wernicke surface-R = C + D + E + F + G + H + I + J;
Wernicke volume-L = C + D + E + F + G + H + I + J;
Wernicke volume-R = C + D + E + F + G + H + I + J.

### Statistical analysis

All statistical analyses were performed with SAS 9.4.

Within-pair analyses were run in four substeps. We first examined possibly confounding demographic differences between females and males. Statistical comparisons between the sex were conducted using the chi-square test for categorical variables (left-handed patients) and Student's *t*-test for continuous variables (age). As no significant differences were found, the left-handed patients were pooled together with the groups. Then, sex differences in language ability assessments were tested for in Student's *t* and Fisher's tests, which suggests that females had poorer language ability compared to males with ASDs. Furthermore, we tested the sex differences between the brain structures of Broca and Wernicke areas (including thickness, surface, and volume). Total Broca and Wernicke volumes were adjusted for when assessing the surface area, but not thickness, and age was adjusted for in all analyses. Finally, to explore whether sex differences in language ability are attributed to the imbalanced development of Broca and Wernicke brain structures, Pearson's and ANOVA tests were used to analyze the correlation between brain structure and language ability assessments. A Bonferroni correction for multiple comparisons was applied.

## Results

### Participant characteristics

The study initially included 47 cases of ASD, of which 3 were excluded due to lack of clinical data. Of the 44 patients, there were 36 males (81.8%) and 8 females (18.2%); the age range was 24–72 months; the GDS language DQ was 18.66–95.5; and among them, there were 14 (31.8%), 24 (54.5%), and 6 (13.6%) cases of grade 2, grade 3, and grade 4 according to the CARS language communication classification, respectively. No significant difference was observed in the analysis of correlation between age and language proficiency assessments, including GDS language DQ (*r* = −0.199, *p* = 0.195) and CARS language communication classification (*F* = 0.080, *p* = 0.923, respectively). In addition, the age (*t* = 0.301, *p* = 0.765)) and percentage of left-handed patients (χ^2^ = 1.105, *p* = 0.334) were also similar among females and males. The demographic data of the participants are shown in Table 1.

**Table 1:** Demographic database of patients.

Total patients of ASD	44
Sex (n/%)	
Female	36 (81.8)
Male	71 (18.2)
Age at ASD (months):	24–72
Range of GDS language DQ:	18.66–95.5
CARS language communication classification (n/%)	
Grade 2	14 (31.8)
Grade 3	24 (54.5)
Grade 4	6 (13.6)

### Sex and language proficiency assessments

**Figure 2:**
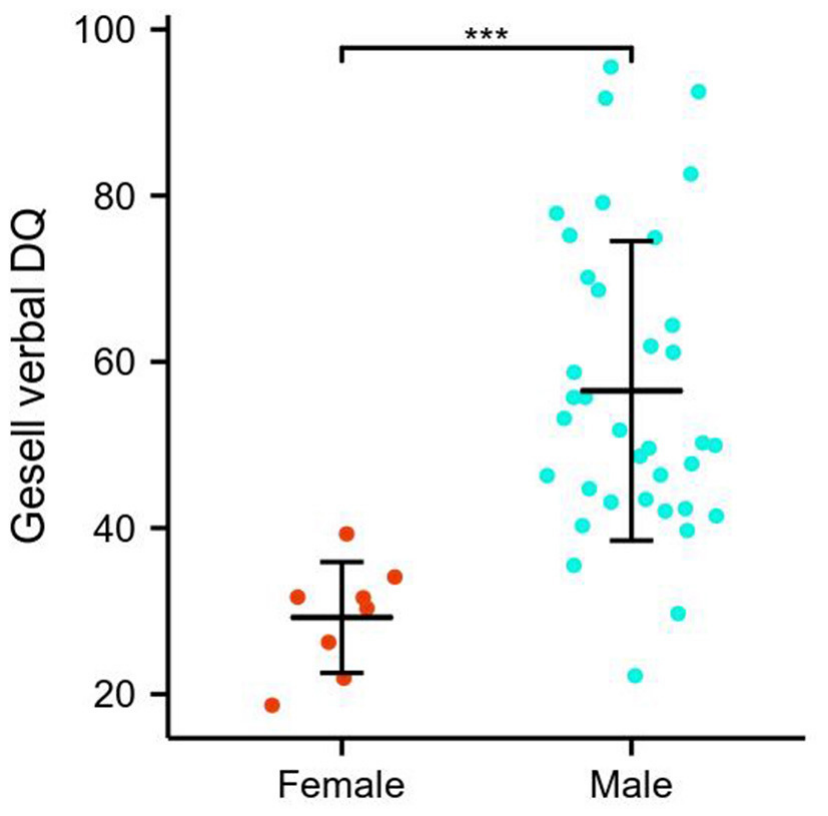
Sex differences of Gesell language DQ in ASDs. ***p < 0.001.

Simple effects for language proficiency assessments and sex differences are detailed in Figure 2 and Table 1. Specifically, the GDS language DQ was significantly higher in males relative to females in ASDs (56.50 ± 18.02 vs. 29.23 ± 6.67, *p* < 0.001). The difference in the distribution of CARS language communication classification between males and females was statistically significant, with females more frequently presenting high grade. There was no significant left-handed sex interaction.

### Sex and Broca and Wernicke areas

**Figure 3:**
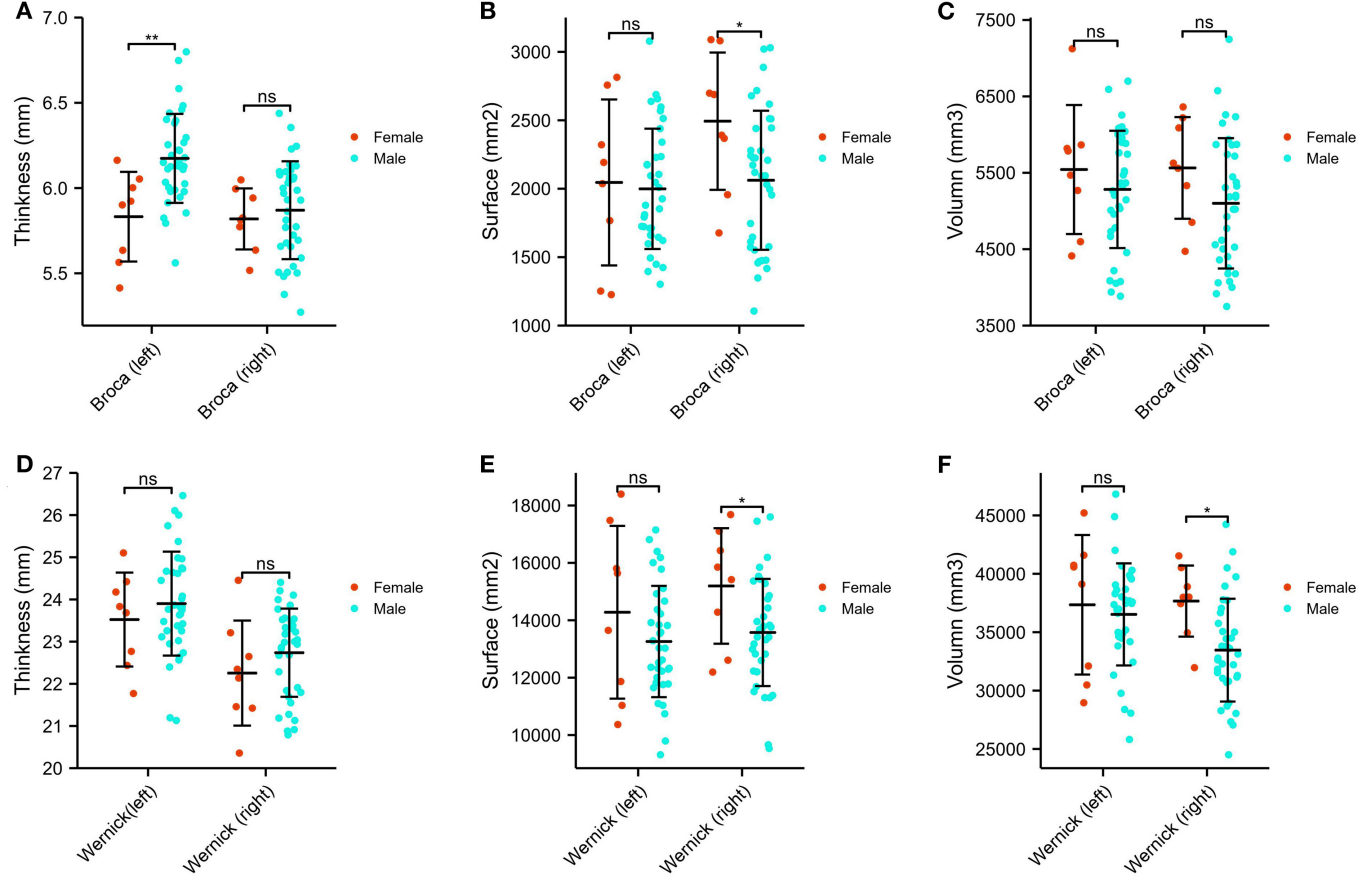
Sex differences of Broca and Wernicke areas in ASDs. **(A)** The comparison of the thickness of the bilateral Broca area. **(B)** The comparison of the surface of the bilateral Broca area. **(C)** The comparison of the volume of the bilateral Broca area. **(D)** The comparison of the thickness of the bilateral Wernicke area. **(E)** The comparison of the surface of the bilateral Wernicke area. **(F)** The comparison of the volume of the bilateral Wernicke area. *p < 0.05, **p < 0.01, ns *p* > 0.05.

Brain structures that differ by sex include the Broca area and Wernicke area (thickness, surface, and volume), and differences are depicted in Figure 3. Specifically, the Broca thickness*-L* is decreased in males relative to females (estimated difference = 3.354, *p* = 0.002), but no sex difference exists in the Broca thickness*-R*. In contrast, the Broca surface*-R*, Wernicke surface*-R*, and Wernicke volume*-R* are significantly decreased in males compared to females (Broca surface*-R*: estimated difference = −2.178, *p* = 0.035; Wernicke surface*-R*: estimated difference = −2.191, *p* = 0.034; Wernicke volume*-R*: estimated difference = −2.555, *p* = 0.014). Again, there was no main effect for being left-handed. In addition, we did not observe sex differences in the Broca surface*-L*, Wernicke surface*-L*, and Wernicke volume*-L*. The study showed that no statistical differences were found in the Broca volume and Wernicke thickness, either left or right, between males and females with ASD.

### Broca and Wernicke brain structures and language ability assessments

To explore whether the differences in language ability between females and males with ASD are due to the imbalanced development of brain structures, Pearson's test was used to analyze the correlation between brain structures and language proficiency assessments. As depicted in Table 2, GSD language DQ was positively correlated with the Broca thickness*-L*, but negatively correlated with the Broca surface*-R*, Wernicke surface*-R*, and Wernicke volume*-R* (all *p* < 0.05). There were only marginally negative correlations between GSD language DQ and Wernicke surface*-L* (*p* = 0.077).

**Table 2:** Sex differences of CARS language communication classification.

	**Female (*n =* 8)**	**Male (*n =* 36)**	**χ^2^**	***P*** **-value**
**CARS language communication (** ***n*** **/%)**				
Grade 2	1 (12.5)	13 (36.1)	8.376^‡^	0.008
Grade 3	3 (37.5)	21 (58.3)		
Grade 4	4 (50.0)	2 (5.6)		

‡ *Fisher's test*.

Although CARS language communication classification contained grades 2–4, there was a significant difference with regard to the Broca thickness*-L* and Broca surface*-R* in all groups (Table 3). Through a two-by-two comparison of brain structures differences between CARS language communication grade, we found that the Broca surface*-R* in grade 4 was higher than that in grades 2 and 3 (2,706.80 ± 340.95 vs. 1,947.73 ± 467.50 and 2,091.18 ± 550.35 [mm^2^], both *p* < 0.05), but the Broca thickness*-L* (5.76 ± 0.17 vs. 6.21 ± 0.30 and 6.11 ± 0.35, both *p* < 0.05) and Wernicke thickness*-R* (21.61 ± 1.13 vs. 22.80 ± 1.01 and 22.65 ± 1.10, both *p* < 0.05) were lower than that in grades 2 and 3.

**Table 3:** Correlation analysis of Broca, Wernicke structures and GDS language DQ.

**Areas**	**GDS language DQ**	
	***r***	***P*** **-value**
**Broca area**		
Thickness (left)	0.382	**0.011**
Thickness (mirror)	0.214	0.164
Surface (left)	-0.22	0.151
Surface (mirror)	-0.322	**0.033**
Volume (left)	0.062	0.690
Volume (mirror)	-0.047	0.760
**Wernicke area**		
Thickness (left)	0.251	0.100
Thickness (mirror)	0.057	0.713
Surface (left)	-0.27	0.077
Surface (mirror)	-0.359	0.017
Volume (left)	-0.186	0.227
Volume (mirror)	-0.343	**0.023**

*Bold values means p < 0.05*.

## Discussion

To the best of our knowledge, this is the first study to analyze the correlation between sex differences in language ability and the Broca/Wernicke area of preschool children with ASD. Females with ASD had significantly lower language ability than males with ASD, and this difference may be due to unbalanced development of certain structures in Broca area and Wernicke area, particularly the Broca thickness*-L*, Broca surface*-R*, Wernicke surface*-R*, and Wernicke volume*-R*.

Many scholars in the past have attributed sex differences and differences in the severity of clinical symptoms to social factors, leading to a long history of neglect for women with ASD. A recent study concluded that sex*-r*elated biological factors (e.g., hormones, genes) may play a role in ASD etiology and have been shown to influence neurodevelopmental trajectories (23). We found that females have a better language development than males at the same preschool age. Females with ASD tend to be at a more severe level by the time it is noticed. Analysis of the relevant brain regions found that this was higher in males than in females, but this sex difference did not exist in Broca thickness*-R*. As Knaus et al. (24) in 2008 also found stronger activation in the Broca area in males with ASD than in controls with typical development, we also suggested that the greater number of white matter fibers in the Broca area of the dominant hemisphere and the stronger connectivity of the various brain regions in males may also be a factor in the better language ability in males.

Human beings have gradually developed language through the accumulation of labor experience and communication, and the activities of hearing words, producing them, reading them, and writing them have led to the development of the corresponding language center in the cerebral cortex. Brodmann's map is one of the best-known maps of the human cerebral cortex, suggesting that the language center is located in four main areas, namely, the primary motor language center, the writing center, the primary auditory language center, and the visual language center. Broca area in the forebrain and Wernicke area in the hindbrain are currently recognized as the main brain areas responsible for linguistic information processing. Broca area was introduced in the nineteenth century by the French surgeon Pierre Paul Broca and was a great challenge to the previous idea of functional holism of the brain; Broca identified the sulcus, the gyrus, as the marker of the functional partition of the brain. Damage to Broca area produces motor aphasia, where they know what they want to say but have difficulty pronouncing it. Wernicke area was introduced in 1874 by the German physician Wernicke. Damage to Wernicke area produces sensory aphasia, which causes dual difficulties in understanding and producing language. This article focuses on Broca area and Wernicke area.

There are no systematic studies to analyze the correlation between language ability and the anatomical structure of brain regions. Both Knaus (10) and Lai (15) analyzed cortical volumes of different brain regions in language studies of ASD. Jäncke (20) analyzed cortical thickness and cortical surface of region of interest, but did not include cortical volume. In this study, cortical volume, thickness, and surface area were all included.

Studies on brain areas associated with ASD have also reported inconsistent results. The relationship between language ability and auditory and Broca/Wernicke area has been corroborated by several studies comparing normal controls with bilinguals (25), regular interpreters (26, 27), simultaneous interpreters (28, 29), or others with particularly strong language abilities (30). Differences in the anatomy of the temporal plane have been found in ASDs with poorer language abilities (31). However, it has also been reported that there are no differences in cortical volumes between the Broca area and Wernicke area in ASDs (32). It has even been suggested that there are structural differences in the Hirschsprung's gyrus at different levels of language ability, even in people without language impairment (33).

In this study, we analyzed the structure of the Broca area and language ability and found that the GDS language DQ was positively correlated with the cortical Broca thickness*-L*, and that children with ASD who had a CARS language level of 4 had a smaller Broca thickness*-L* than those who had a language level of 2 and 3, combined with a higher DQ score in the GDS indicating better language ability and CARS, as showen in Table 4. This means that the greater the Broca thickness*-L*, the better the language ability demonstrated by the GDS and CARS. The Broca surface*-R* is negatively correlated with the GDS language DQ, with children with a CARS language level of 4 having a higher surface area than children with a language level of 2 and 3, which means that the smaller the Broca surface*-R*, the better the language ability. The correlation between the structure of Broca area and its right area and language ability suggests that there is a lateralization of Broca area in children with ASD, with children with better language ability having increased cortical thickness in the dominant hemisphere of Broca area and reduced surface area in the right area, confirming the theory of language lateralization.

**Table 4:** Differential analysis of CARS language communication classification and brain structures.

**Areas**	**Grade 2 (*****n** =* **14)**	**Grade 3 (*****n** =* **24)**	**Grade 4 (*****n** =* **6)**	***F***	***P*** **-value**
**Broca area**					
Thickness (left), mm	6.21 ± 0.30	6.11 ± 0.35	5.76 ± 0.17^a^^b^	3.422	0.042
Thickness (right), mm	5.88 ± 0.45	5.85 ± 0.28	5.71 ± 0.15	0.495	0.613
Surface (left), mm^2^	1,905.36 ± 523.39	2,012.68 ± 449.63	2,268.00 ± 407.57	1.047	0.360
Surface (right), mm^2^	1,947.73 ± 467.50	2,091.18 ± 550.35	2,706.80 ± 340.95^a^^b^	3.919	0.028
Volume (left), mm^3^	5,186.00 ± 1,031.23	5,375.71 ± 647.46	5,322.20 ± 997.34	0.225	0.800
Volume (right), mm^3^	4,760.09 ± 1,201.27	5,207.57 ± 713.28	5,559.60 ± 1,181.57	1.576	0.219
**Wernicke area**					
Thickness (left), mm	23.90 ± 1.142	23.77 ± 1.25	23.61 ± 0.92	0.102	0.903
Thickness (right), mm	22.80 ± 1.01	22.65 ± 1.10	21.61 ± 1.13^a^^b^	2.236	0.120
Surface (left), mm^2^	12,866.18 ± 2,353.56	13,549.00 ± 2,241.79	13,980.40 ± 1,447.95	0.556	0.578
Surface (right), mm^2^	13,068.10 ± 2,037.76	14,049.57 ± 1,941.78	15,365.80 ± 836.07^a^	2.645	0.083
Volume (left), mm^3^	47,331.82 ± 6,694.99	49,225.61 ± 7,089.30	50,391.20 ± 7,715.64	0.413	0.665
Volume (right), mm^3^	42,820.36 ± 5,719.88	45,218.29 ± 5,409.70	44,259.40 ± 4,267.67	0.788	0.461

“a” *indicates statistically significant compared to grade 2 (P < 0.05),*

“b” *indicates statistically significant compared to grade 3 (P < 0.05)*.

In our study of Wernicke area and language ability, we found that GDS language DQ did not correlate significantly with cortical thickness, surface, and volume in the left Wernicke's area, and that Wernicke surface*-R* and Wernicke volume*-R* were negatively correlated. This suggests that children with ASD with different language ability do not have significant structural differences in the dominant hemisphere of the Wernicke area, whereas children with ASD with poor language ability show structural reductions in cortical thickness, surface, and volume in the right areas of the nondominant hemisphere. Therefore, our study should not only focus on the dominant hemisphere, but also on the nondominant hemisphere. In addition, because of the coexistence of synergistic and competitive mechanisms between the functions of the left and right hemispheres, we hypothesized that children with ASD may still be in a state of inhibition in the nondominant hemisphere when the nondominant hemisphere appears to have reduced structure, while dominance and activation in the dominant hemisphere are still in existence, and therefore giving timely and targeted language training may lead to recovery and catch-up of language ability in children with ASD, which explains the previously reported phenomenon of some children with ASD showing language delays early on, but catching up with their peers by school age.

This study on language ability and the structure of Broca and Wernicke areas in children with ASD found that the differences in language ability in children with ASD were mainly in Broca area, while the structural differences in Wernicke area were not significant. Damage to Broca area, the motor language center, which is responsible for the processing of linguistic information and the production of language, including the production of coordinated articulatory procedures, the organization of the grammatical structure of language, and the generation of motivation and desire for speech, results in motor aphasia, where the person knows what is on their mind but cannot express it. Sensory aphasia, a dual impairment of speech comprehension and speech production, is typical in that the person speaks fluently but without any logic. Therefore, we hypothesized that the language problems of children with ASD may be due to abnormalities in the expression and production of language, but not in the processing and comprehension of language.

Due to the complexity of language production, processing, transmission, and expression, there is currently a lack of standardized and unified language testing instruments worldwide. The next step is to conduct a multicenter study to expand the sample size and seek more authoritative language evaluation indicators, with the aim of locating the language of ASD in the specific aspects of information processing, transmission, and expression. We will continue to explore the synergistic relationship between language ability and structural/functional connectivity in Broca and Wernicke areas by fMRI.

## Conclusion

There are sex differences in the language abilities of preschool children with ASD, with females with ASD having significantly lower language abilities than males with ASD, and this difference may be due to an imbalance in the development of certain structures in Broca area and Wernicke area, more closely related to the structure of Broca area. Children with ASD may not have abnormalities in the processing and comprehension of language, but may have abnormalities in the expression and production of language. We expect early identification, early diagnosis, early instruction, and intervention to improve language ability through MR examinations.

## Data availability statement

The raw data supporting the conclusions of this article will be made available by the authors, without undue reservation.

## Ethics statement

The studies involving human participants were reviewed and approved by Institutional Research Ethics Board of Chongqing Children's Hospital (IRB No. [2018] Ethical Review [Research] No. [82]). Written informed consent to participate in this study was provided by the participants' legal guardian/next of kin.

## Author contributions

All authors listed have made a substantial, direct, and intellectual contribution to the work and approved it for publication.

## Funding

This study was funded by the National Nature Science of Foundation of China (81770526 and 81771223) and Intelligent Medicine Research Project of Chongqing Medical University in 2020 (Grant No. ZHYX202030) for data collection.

## Conflict of interest

The authors declare that the research was conducted in the absence of any commercial or financial relationships that could be construed as a potential conflict of interest.

## Publisher's Note

All claims expressed in this article are solely those of the authors and do not necessarily represent those of their affiliated organizations, or those of the publisher, the editors and the reviewers. Any product that may be evaluated in this article, or claim that may be made by its manufacturer, is not guaranteed or endorsed by the publisher.

## Abbreviations

ASD: autism spectrum disorder
DQ: development quotient
GDS: Gesell Developmental Scale
CARS: Childhood Autism Rating Scale
SRS: Social Responsiveness Scale
ABC: Autism Behavior Checklist
ADOS-2, Autism Diagnostic Observation Schedule: Second Edition
Broca thickness*-L*: left Broca cortical thickness
Broca thickness*-R*: right Broca cortical thickness
Broca surface*-L*: left Broca cortical surface
Broca surface*-R*: right Broca cortical surface
Broca volume*-L*: left Broca cortical volume
Broca volume*-R*: right Broca cortical volume
Wernicke thickness*-L*: left Wernicke cortical thickness
Wernicke thickness*-R*: right Wernicke cortical thickness
Wernicke surface*-L*: left Wernicke cortical surface
Wernicke surface*-R*: right Wernicke cortical surface
Wernicke volume*-L*: left Wernicke cortical volume
Wernicke volume*-R*: right Wernicke cortical volume
DC: degree centrality.
